# An Eye Tracking Analysis on Diagnostic Performance of Digital Fundus Photography Images between Ophthalmologists and Optometrists

**DOI:** 10.3390/ijerph17010030

**Published:** 2019-12-18

**Authors:** Mizhanim Mohamad Shahimin, Azalia Razali

**Affiliations:** 1 Optometry & Vision Sciences Program, Faculty of Health Sciences, Universiti Kebangsaan Malaysia, Kuala Lumpur Campus, Jalan Raja Muda Abdul Aziz, Kuala Lumpur 50300, Malaysia; 2 Ophthalmology Department, Hospital Selayang, Lebuhraya Selayang-Kepong, Batu Caves, Selangor 68100, Malaysia; azaliarazali@gmail.com

**Keywords:** eye movements, eye tracker, fundus photographs, diagnosis, diabetic retinopathy

## Abstract

To investigate the parameters of eye movement between ophthalmologists and optometrists while diagnosing digital fundus photographs, sixteen participants (eight ophthalmologists and eight optometrists) were recruited in this study. Every participant’s eye movement during diagnosis of a randomized set of fundus photographs displayed on an eye tracker were recorded. Fixation metrics (duration, count and rate) and scan path patterns were extracted from the eye tracker. These parameters of eye movement and correct diagnosis score were compared between both groups. Correlation analyses between fixation metrics and correct diagnosis score were also performed. Although fixation metrics between ophthalmologists and optometrists were not statistically different (*p* > 0.05), these parameters were statistically different when compared between different area of interests. Both participant groups had a similar correct diagnosis score. No correlation was found between fixation metrics and correct diagnosis score between both groups, except for total fixation duration and ophthalmologists’ diagnosis score of diabetic retinopathy photographs. The ophthalmologists’ scan paths were simpler, with larger saccades, and were distributed at the middle region of the photographs. Conversely, optometrists’ scan paths were extensive, with shorter saccades covering wider fundus areas, and were accumulated in some unrelated fundus areas. These findings indicated comparable efficiency and systematic visual search patterns between both the groups. Understanding visual search strategy could expedite the creation of a novel training routine for interpretation of ophthalmic diagnostic imaging.

## 1. Introduction

Digital fundus photography is routinely performed by eye care practitioners, as they complement the evaluation, diagnosis and subsequent management of eye diseases [1]. Fundus photographs are also used to explore visual perception and visual cognition in the process of making diagnoses from the photographs [2]. The method of fundus photograph examination and the underlying cognitive process during the diagnosis procedure can be revealed through an eye tracking application [3,4,5]. An eye tracker objectively records and provides identification of the relevant fundus’ areas of interest, as revealed by fixation in eye movement. Fixation is a period when the eye remains stationary to gather visual information. High fixation duration or count on a particular area indicates high attention to the area. Saccades are ballistic eye movements between two fixations in which no visual information is obtained during the process. A scan path is a complete saccade–fixate–saccade sequence and is beneficial in tracing overall eye movements through a scene or images [6].

In recent years, the roles of optometrists at hospitals and primary health care centers have been extended to screening diabetic retinopathy through fundus photography [7]. Optometrists are responsible for grading the disease and deciding on the proper management of each case as the forefront for primary eye care providers, especially in remote areas, where ophthalmologists are unavailable. During the screening process, incidental findings of other ocular diseases are frequent, and optometrists have a shared-care role with ophthalmologists to help in detecting and providing referrals for the particular disease. This condition requires appropriate knowledge and systematic examination of the fundus photographs to identify characteristic features of pertaining diseases. Therefore, eye tracking is useful for the development of eye movement profiles of ophthalmologists and optometrists during fundus photographs diagnosis. Both ophthalmologists and optometrists were selected to represent the normal scenario, reflecting the clinical scope of work for senior eye care professionals that involves a final decision in diagnosing patients. This will facilitate the understanding of differences in performances between these two key eye care practitioners and aid improvement in diagnostic performance through visual search behavior analysis.

The aim of this paper was to investigate parameters of eye movement when ophthalmologists and optometrists diagnose normal and diabetic retinopathy digital fundus images. First, we presented a randomized set of digital fundus images to research participants and then recorded their eye fixation positions and scan path using the Tobii TX300 eye tracker. Next, we identified the area of interest (AOI) in each image and compared the fixation duration, fixation count, and ratio of fixation count to fixation duration at the AOI between ophthalmologists and optometrists. Finally, we determined whether the parameters of eye movements correlate with the diagnostic accuracy, which has clinical implications on the treatment and management of patients.

## 2. Materials and Methods

This experimental study involved sixteen participants, comprising ophthalmologists and optometrists from a public hospital. Eight experienced and board-certified ophthalmologists (mean age = 38.38 ± 2.20 years) and eight optometrists with more than 5 years of clinical working experience (mean age = 33.88 ± 3.56 years) were purposively sampled and were naïve to the eye tracking protocols. All participants were informed about the experimental procedures and consented to the study. Ethical approval was obtained from the Universiti Kebangsaan Malaysia’s Research Ethics Committee (Approval no.: UKM 1.5.3.5/244/NN-069-2015).

All participants were required to diagnose a randomized set of twelve digital fundus photographs (six photographs were diabetic retinopathy images and another six were of normal fundus appearance) that were projected on the Tobii TX300 eye tracker’s 23 inch monitor screen with a resolution of 1920 × 1080 pixels (Tobii Technology AB, Stockholm, Sweden). The eye tracker used was non-invasive and utilizes near infrared light to track eye movements. The Tobii TX300 has a sampling rate of 300 Hz with an accuracy of 0.4°.

The eye tracking protocols began with a calibration process to ensure that the eye tracker is able to detect precise eye movements before the fundus photograph diagnosis process begins. Participants were seated at 60 cm away from the eye tracker screen and a chin rest was used to ensure the participants’ head movement remained within the eye tracker measuring area. Each participant then underwent an automated nine-point calibration test that extends over the eye tracker’s monitor screen. This calibration test provides immediate participant-specific data point accuracy. Participants were instructed to view twelve digital fundus photographs displayed on the eye tracker monitor and were asked to diagnose each photograph verbally, either of diabetic retinopathy or healthy appearance. No details of patient medical history, ophthalmic investigations or other test results were given to any participant. The 12 images were presented sequentially in the same random order pre-set on the Tobii Studio™ analysis software for every participant. There was no time limit for the diagnosis process in order to mimic the real diagnosis process and participants were able to control the presentation of the fundus photograph by mouse clicks. Participants’ eye movements were recorded simultaneously while the diagnosis process took place.

**Figure 1:**
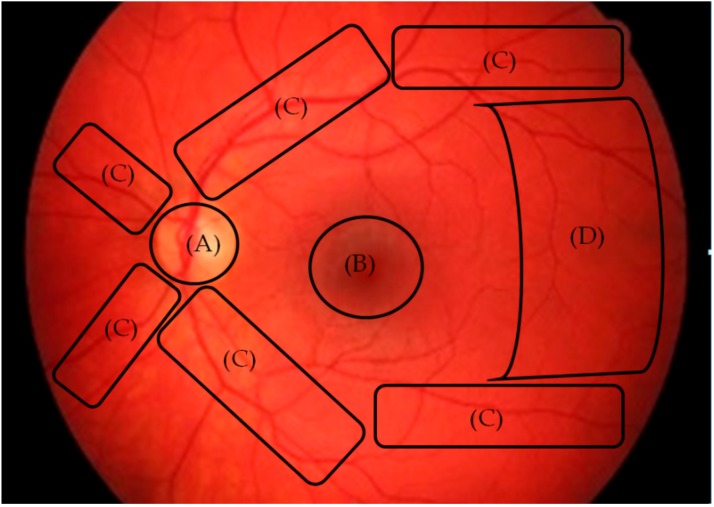
Illustration of area of interest (AOI) for each fundus photograph, covering the optic disc (**A**), macula (**B**), major blood vessels (**C**) and retina region (**D**).

Parameters of eye movement consist of quantitative data (fixation duration and fixation count) and qualitative data (scan path patterns) were extracted from the eye tracker. Quantitative data were derived during post-analysis from a predetermined area of interest (AOI) that was assigned on each fundus photograph. Fundus anatomical landmarks such as the optic disc, macula, major blood vessels, lesions/abnormality area and characteristic features pertaining to diabetic retinopathy were included as the AOI (Figure 1). The fixation rate was also derived by calculating the ratio of the fixation count to fixation duration per AOI [8]. Qualitative data or the scan path patterns were examined and describe subjectively by analyzing the direction and length of the eye movement from one location to the next and subjectively looking at the scan path patterns. Participants’ diagnosis outputs for every fundus photograph were recorded during the eye tracking procedure for further analysis.

## 3. Results

### 3.1. Quantitative Analysis

The quantitative analysis included a comparison of the mean fixation duration, count and rate and the diagnosis accuracy score between ophthalmologists and optometrists. Correlation tests were also performed to investigate the relationships between accurate diagnosis scores (percentage) and fixation metrics (duration, count and rate) across a group of fundus photographs for both participants. The AOIs for healthy fundus photographs were grouped consisting of the disc, macula, retina areas and major blood vessels, while the AOIs for diabetic retinopathy fundus photographs include the disc, macula and the specific lesions and abnormality areas (laser scar, hemorrhage and cotton wool spots).

#### 3.1.1. Fixation Duration

Descriptive analysis showed that ophthalmologists’ fixation duration ranges from 0.38 to 16.35 msec, while optometrists’ fixation duration ranges from 0.65 to 16.65 msec. Participants’ fixation duration was longer in the optic disc area of healthy digital fundus images compared to the same area of diabetic retinopathy digital fundus images. This was followed by the macula area, retina, major blood vessels and other lesions or abnormality areas. The most fixated lesion or abnormality area was the hemorrhage areas of the diabetic retinopathy digital fundus images (Table 1).

We performed one-way ANOVA analysis to determine whether there are any statistically significant differences between the mean fixation duration of the AOIs between ophthalmologists and optometrists (Table 2). Analysis revealed that there were statistically significant differences between AOI groups of healthy fundus photographs for ophthalmologists (F (3,28) = 21.52, *p* ≤ 0.001) and for optometrists (F (3,28) = 36.03, *p* ≤ 0.001). Tukey post hoc tests also revealed that the mean fixation durations on the AOIs were statistically significant between all AOIs (*p* ≤ 0.001) for both participant groups (Table 3).

Analysis on diabetic retinopathy fundus photographs also showed statistically significant differences between mean fixation duration on different AOIs for ophthalmologists (F (4,35) = 7.60, *p* ≤ 0.001) and optometrists (F (4,35) = 10.30, *p* ≤ 0.001) (Table 2). Pairwise comparisons of the means using Tukey post hoc tests indicated only two significant comparisons for ophthalmologists, which was the AOI of laser scar (mean difference = 3.37, *p* ≤ 0.001) and the AOI of cotton wool spots (mean difference = 3.67, *p* ≤ 0.001). The other comparisons were not statistically significant (*p* > 0.05). The Tukey post hoc comparison for optometrists revealed three pairwise significant comparisons for macula AOI (mean difference = 3.89, *p* ≤ 0.001), laser scar AOI (mean difference = 4.17, *p* ≤ 0.001) and cotton wool spots AOI (mean difference = 4.65, *p* ≤ 0.001) (Table 4).

#### 3.1.2. Fixation Count

Descriptive analysis of fixation count showed that the fixation count for ophthalmologists and optometrists was highest at the optic disc (healthy fundus images) and was lowest at the cotton wool spots area. The fixation count for ophthalmologists was higher than the optometrists for healthy fundus photographs (optic disc and macula) but was lower for the diabetic retinopathy fundus photographs (optic disc) (Table 5).

There were statistically significant differences for the mean fixation count between AOIs of healthy fundus photographs as demonstrated by one-way ANOVA for ophthalmologists (F (3,28) = 22.28, *p* ≤ 0.001) and for optometrists (F (3,28) = 23.35, *p* ≤ 0.001) (Table 6). The mean fixation count for diabetic retinopathy photographs was also unequal according to the one-way ANOVA for the ophthalmologists, F (4,35) = 9.55, *p* ≤ 0.001) and for optometrists, F (4,35) = 13.43, *p* ≤ 0.001) (Table 6).

Tukey post hoc tests showed that the mean fixation count on the pairwise AOIs was statistically significant (*p* ≤ 0.001) between all AOIs of healthy fundus photographs as presented in Table 7 for both ophthalmologists and optometrists.

Tukey post hoc tests were also performed on the mean fixation count for the pairwise AOI comparisons for diabetic retinopathy photographs. The results revealed two statistically significant pairwise comparisons, which were on laser scar AOI (mean difference = 10.13, *p* ≤ 0.001) and on the cotton wool spot AOI (mean difference = 11.38, *p* ≤ 0.001) for the ophthalmologists. The pairwise AOI comparisons for the optometrists showed significant results for all AOIs as presented in Table 8.

#### 3.1.3. The Fixation Rate

The fixation rate was analyzed based on the predetermined AOIs by calculating the ratio of fixation count over fixation duration. Table 9 shows that the fixation rate for ophthalmologists ranges from 1.83 to 4.76 and was between 1.44 and 5.06 for optometrists. The highest fixation rate was recorded at the major blood vessels area for ophthalmologists and macula area (diabetic retinopathy fundus images) for optometrists. This implies that both groups spent more time inspecting the respective AOIs. Furthermore, both groups had the lowest fixation rate at the cotton wool spots lesion area, suggesting that they have lower efficiency in search tasks at this AOI.

One-way ANOVA analyses performed on the fixation rate for both participant groups revealed that there were no statistically significant differences between AOI groups except for the optometrists when diagnosing healthy fundus images (F (3,28) = 5.16, *p* = 0.01) (Table 10).

A further Tukey post hoc test was performed on the mean fixation rate for the pairwise AOI comparisons of the significant finding for optometrists (Table 11). The results revealed two statistically significant pairwise comparisons which were on the macula AOI (mean difference = −1.75, *p* = 0.02) and on the retina areas AOI (mean difference = −1.98, *p* = 0.01) only.

#### 3.1.4. Diagnosis Accuracy

Descriptive analysis showed that both groups performed better in diagnosing diabetic retinopathy fundus photographs (mean score (ophthalmologists) = 81.25 ± 10.68; mean score (optometrists) = 87.50 ± 11.79) compared to healthy fundus photographs (mean score (ophthalmologists) = 32.29 ± 27.97; mean score (optometrists) = 37.50 ± 27.82). The ophthalmologists achieved a higher percentage of total correct diagnosis (66.67%) compared to optometrists (62.50%) (Table 12).

Paired t-tests were conducted to compare the correct diagnosis between the ophthalmologists and optometrists for healthy and diabetic retinopathy fundus photographs, as well as the total correct diagnosis scores (Table 13). There were no statistically significant differences in the scores between groups for healthy fundus photographs (mean difference = 0.88 ± 2.03, *t* (7) = 1.22, *p* = 0.26), for diabetic retinopathy fundus photographs (mean difference = −0.38 ± 1.19, *t* (7) = −0.89, *p* = 0.40) and for the total correct diagnosis score (mean difference = 0.50 ± 1.77, *t* (7) = 0.80, *p* = 0.45). This showed that optometrists had equal performance as the ophthalmologists in making correct diagnosis for healthy and diabetic retinopathy fundus photographs.

#### 3.1.5. Correlation between Fixation Metrics (Duration, Count and Rate) and Correct Diagnosis

We performed Pearson’s Correlation tests to investigate any relationships between total fixation duration, count and rate to the correct diagnosis scores. Results of the Pearson’s correlation indicated that there was a significant positive association between correct diagnosis for diabetic retinopathy fundus images and ophthalmologists’ total fixation duration, (*r* = 0.77, *p* = 0.03) (Table 14). However, no correlation was found for other parameters tested.

### 3.2. Qualitative Analysis

**Figure 2:**
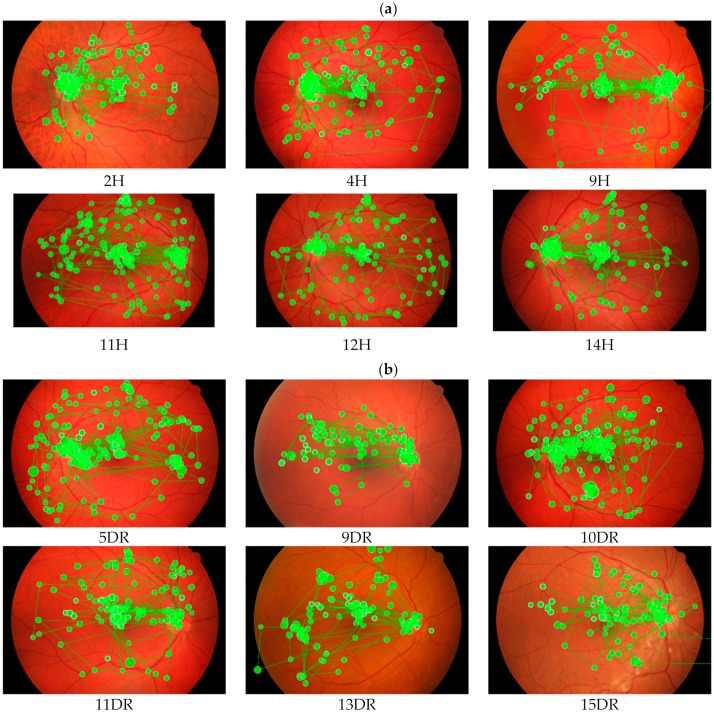
A sample of scan path pattern from an ophthalmologist when inspecting and diagnosing healthy and diabetic retinopathy digital fundus photographs. (**a**) Healthy digital fundus photographs (coded as 2H, 4H, 9H, 11H, 12H and 14 H); (**b**) Diabetic retinopathy digital fundus photographs (coded as 5DR, 9DR, 10DR, 11DR, 13DR and 15DR).

Qualitative inspection of ophthalmologists’ scan path showed that their visual scanning patterns were simpler compared to optometrists. They had a larger visual span to gain information across a wider field of vision with a lower number of fixations. In general, their chronological scan path sequence was macula–disc–macula–retina. The inferior and nasal retinae were the less inspected region. The visual coverage distributed more at the middle region of the photographs and rarely extended beyond the posterior pole region. However, their visual coverage would cover a wider area with a higher fixation count if no prominent features could be identified. This pattern of fixation was apparent when viewing the healthy fundus photographs (Figure 2).

**Figure 3:**
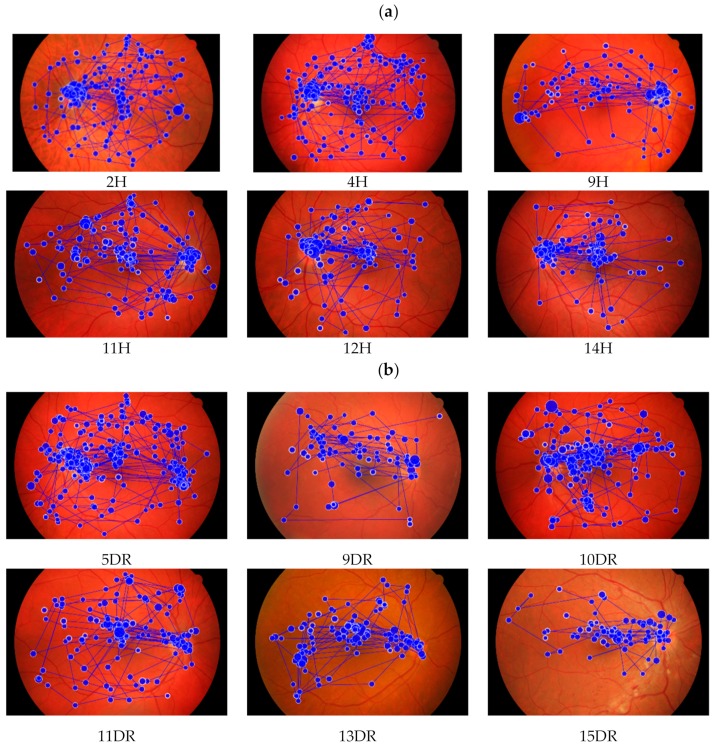
A sample of scan path pattern from an optometrist when inspecting and diagnosing healthy and diabetic retinopathy digital fundus photographs; (**a**) Healthy digital fundus photographs (coded as 2H, 4H, 9H, 11H, 12H and 14 H); (**b**) Diabetic retinopathy digital fundus photographs (coded as 5DR, 9DR, 10DR, 11DR, 13DR and 15DR).

On the contrary, optometrists’ visual coverage was more extensive without a particular pattern or sequence (Figure 3). Collectively, their visual distribution was higher and covered the middle and the peripheral fundus region. The saccades were noticeably shorter between fixations. These patterns of observation were apparent when optometrists were diagnosing healthy fundus photographs. In contrast their visual coverage was lower during diabetic retinopathy photographs viewing with lower number of fixations. The scan path patterns were almost similar with ophthalmologists’ (photo 9DR, 11DR, 13DR and 15DR).

## 4. Discussion

Analysis showed that the fixation metrics (duration, count and rate) between ophthalmologists and optometrists were not statistically different during diagnosis of healthy and diabetic retinopathy fundus photographs (*p* > 0.05). However, the fixation duration per different AOI was significantly different for both participant groups for both healthy and diabetic retinopathy fundus photographs (*p* < 0.05). A similar pattern was also documented for fixation count. Both ophthalmologists and optometrists fixated more on important fundus anatomical landmarks (optic disc, macula and blood vessels), compared to other structures. The optic disc inspection is important, as this is the obvious feature that will exhibit possible changes in ocular diseases such as the appearance of new vessels in diabetic retinopathy [9]. Examining macula and the major blood vessels will ascertain whether there are any irregularities in the structures that indicate ocular diseases. Moreover, fundus examination is always taught to be performed systematically during training by examining the fundus according to the anatomical landmarks to avoid any missed fundus changes [9,10]. This could explain why both participants spent more time looking at the fundus features (optic disc, macula and blood vessels) compared to other structures. Low fixation metrics on the lesions and other abnormalities such as the laser marks, hemorrhage and cotton wool spots may imply that the abnormalities were too obvious that they did not fixate at the same AOI longer. However, this assumption can only be confirmed if retrospective think aloud method was applied during data collection, in which participants would verbalize their thoughts after a playback of their recording [11]. This method could be implemented in the future research for added value in medical imaging interpretation studies.

Although the quantitative data showed non-significant findings, qualitative data indicated that ophthalmologists had better systematic visual scan patterns compared to optometrists. This could be explained by their perceptual ability to chunk together groups of relevant features, rather than individual features. This may also imply that the ophthalmologists process information from a larger segment of the fundus resulting in larger visual sweeps. They make use of parafoveal and peripheral processing to extract information from a wider region of the fundus during fixation. This also allows a global impression of the digital fundus photograph content from the initial and few subsequent fixations [12,13,14]. In contrast, optometrists made a shorter visual span by examining the fundus photographs from point to point rather than segments to segments. This showed that they sampled information on a local scale. The advantage of this pattern is that they covered more areas of the fundus and more areas of interest were fixated. At the same, this resulted in more fixations and they took longer time to inspect a fundus photograph [2,12,15,16].

Good diabetic retinopathy diagnosis performance of both groups is maybe associated with the nature of the disease. Retina manifestation of diabetic retinopathy is obvious and is easy to spot regardless of the stage of the disease. This condition facilitates the diagnosis process as the lesions attract the examiner’s attention. Conversely, the theory of visual saliency and knowledge top-down control could provide the basis for easy interpretation of diabetic retinopathy photographs [17,18]. Diagnostic performance of ophthalmologists and optometrists was similar in inspecting diabetic retinopathy digital fundus photographs. Optometrists could have developed this expertise through familiarity with the task. Ophthalmologists’ perceptual expertise is attributed to their more structured and comprehensive training as well as higher case volume experience [2].

The correlation between total fixation metrics and diagnostic accuracy for ophthalmologists for diabetic retinopathy fundus images was positive, with a moderate association strength. The positive correlation indicates that a longer scanning or viewing time correlates with a higher chance of making a correct diagnosis. This is supported by Rangrej et al. (2018), who found a similar finding for fundus diagnosis [19]. However, the association was only true for one parameter in our study. Other parameters showed non-significant findings, implying that there are no associations between the total fixation duration, count and rate and the correct diagnosis. It is interesting to note that a previous study investigating the association between eye movement and diagnostic error in mammography image analysis found that the longer review time correlated with more chances of making a diagnostic error rather than correct diagnosis [20]. An earlier study also reported that a longer gaze duration was associated with a false negative decision than true negative [21].

## 5. Conclusions

Although the quantitative data presented in this study do not show significant findings differentiating the eye movement parameters between ophthalmologists and optometrists, qualitative data showed different scan path patterns of the visual search behavior between ophthalmologists and optometrists. Despite the fact that the sample size for participant groups has been calculated to ascertain the power of the study, a deeper understanding could be obtained if the stimuli (fundus photography images) used were increased. Furthermore, the availability of the eye tracker’s raw data with embedded user-friendly eye tracker software could contribute to the analysis and interpretation of eye tracking data [22]. Future work should concentrate on looking at the effects of learning experience and training in formulating correct diagnosis for fundus image analysis. The use of a mobile eye tracker could be implemented to simulate the real process of diagnosis with fundus photographs.

## Figures and Tables

**Table 1:** Mean fixation duration on different areas of interests (AOIs) between ophthalmologists and optometrists.

Areas of Interest	Mean (msec) ± SD		Mean Difference (msec) ± SD
	Ophthalmologists	Optometrists	
Optic disc (H)	16.35 ± 8.77	16.65 ± 6.48	−0.30 ± 13.58 (NSS)
Optic disc (DR)	3.75 ± 2.13	4.82 ± 3.46	−1.08 ± 4.25 (NSS)
Macula (H)	3.05 ± 1.97	2.59 ± 1.33	−1.08 ± 4.25 (NSS)
Macula (DR)	1.87 ± 2.13	0.93 ± 0.83	0.94 ± 2.49 (NSS)
Retina areas	1.33 ± 0.56	1.94 ± 1.42	−0.61 ± 1.53 (NSS)
Major blood vessels	0.70 ± 0.47	1.45 ± 1.43	−0.76 ± 1.60 (NSS)
Lesions/abnormality area			
-laser scar	0.38 ± 0.41	0.65 ± 0.35	−0.27 ± 0.63 (NSS)
-hemorrhage	2.03 ± 1.47	2.49 ± 1.02	−0.46 ± 2.29 (NSS)
-cotton wool spots	1.83 ± 2.55	1.44 ± 2.39	−0.09 ± 0.29 (NSS)

H = healthy fundus images; DR = Diabetic Retinopathy fundus images; NSS = not statistically significant.

**Table 2:** Analysis of variance (ANOVA) for mean fixation duration on different areas of interests (AOIs) between ophthalmologists and optometrists.

Groups		df	F Value	*p* Value
Ophthalmologists	Between AOIs (H)	3	21.52	<0.001 (SS)
	Within AOIs (H)	28		
Optometrists	Between AOIs (H)	3	36.03	<0.001 (SS)
	Within AOIs (H)	28		
Ophthalmologists	Between AOIs (DR)	4	7.60	<0.001 (SS)
	Within AOIs (DR)	35		
Optometrists	Between AOIs (DR)	4	10.30	<0.001 (SS)
	Within AOIs (DR)	35		

H = healthy fundus images; DR = diabetic retinopathy fundus images; SS = statistically significant.

**Table 3:** The Tukey post hoc analysis of multiple comparisons for mean fixation duration on different areas of interests (AOIs) for healthy fundus photographs between ophthalmologists and optometrists.

Groups	AOI Comparison Areas		Mean Difference	*p* Value
Ophthalmologists	Disc	Macula	13.30	<0.001 (SS)
		Retina Areas	15.02	<0.001 (SS)
		Major Blood Vessels	15.65	<0.001 (SS)
Optometrists	Disc	Macula	14.06	<0.001 (SS)
		Retina Areas	14.70	<0.001 (SS)
		Major Blood Vessels	15.19	<0.001 (SS)

SS = statistically significant.

**Table 4:** The Tukey Post hoc analysis of multiple comparisons for mean fixation duration on different areas of interests (AOIs) for diabetic retinopathy photographs between ophthalmologists and optometrists.

Groups	AOI Comparison Areas		Mean Difference	*p* Value
Ophthalmologists	Disc	Macula	1.88	0.12 (NSS)
		Laser scar	3.37	<0.001 (SS)
		Hemorrhage	1.71	0.18 (NSS)
		Cotton wool spots	3.67	<0.001 (SS)
Optometrists	Disc	Macula	3.89	<0.001 (SS)
		Laser scar	4.17	<0.001 (SS)
		Hemorrhage	2.33	0.06 (NSS)
		Cotton wool spots	4.65	<0.001 (SS)

SS = statistically significant; NSS = not statistically significant.

**Table 5:** Mean fixation count on different areas of interests (AOIs) between ophthalmologists and optometrists.

Areas of Interest	Mean (*n*) ± SD		Mean Difference (*n*) ± SD
	Ophthalmologists	Optometrists	
Optic disc (H)	40.13 ± 19.48	39.75 ± 17.17	0.38 ± 32.10 (NSS)
Optic disc (DR)	11.75 ± 5.23	14.63 ± 8.60	−2.88 ± 9.19 (NSS)
Macula (H)	11.25 ± 5.97	9.88 ± 4.76	1.38 ± 8.14 (NSS)
Macula (DR)	6.38 ± 6.44	3.38 ± 2.45	3.00 ± 7.69 (NSS)
Retina areas	5.88 ± 2.48	8.13 ± 5.22	−2.25 ± 6.27 (NSS)
Major blood vessels	2.50 ± 1.60	4.75 ± 3.99	−2.25 ± 4.59 (NSS)
Lesions/abnormality area			
-laser scar	1.63 ± 1.51	2.75 ± 1.28	−1.13 ± 2.03 (NSS)
-hemorrhage	7.00 ± 4.00	8.25 ± 3.54	−1.25 ± 6.74 (NSS)
-cotton wool spots	0.38 ± 0.52	0.50 ± 0.76	−0.13 ± 0.99 (NSS)

H = healthy fundus images; DR = diabetic retinopathy fundus images; NSS = not statistically significant.

**Table 6:** Analysis of variance (ANOVA) for mean fixation count on different areas of interests (AOIs) between ophthalmologists and optometrists.

Groups		df	F Value	*p* Value
Ophthalmologists	Between AOIs (H)	3	22.28	<0.001 (SS)
	Within AOIs (H)	28		
Optometrists	Between AOIs (H)	3	23.35	<0.001 (SS)
	Within AOIs (H)	28		
Ophthalmologists	Between AOIs (DR)	4	9.55	<0.001 (SS)
	Within AOIs (DR)	35		
Optometrists	Between AOIs (DR)	4	13.43	<0.001 (SS)
	Within AOIs (DR)	35		

H = healthy fundus images; DR = diabetic retinopathy fundus images; SS = statistically significant.

**Table 7:** The Tukey post hoc analysis of multiple comparisons for mean fixation count on different areas of interests (AOIs) for healthy fundus photographs between ophthalmologists and optometrists.

Groups	AOI Comparison Areas		Mean Difference	*p* Value
Ophthalmologists	Disc	Macula	28.88	<0.001 (SS)
		Retina Areas	34.25	<0.001 (SS)
		Major Blood Vessels	37.63	<0.001 (SS)
Optometrists	Disc	Macula	29.88	<0.001 (SS)
		Retina Areas	31.63	<0.001 (SS)
		Major Blood Vessels	35.00	<0.001 (SS)

SS = statistically significant.

**Table 8:** The Tukey post hoc analysis of multiple comparisons for mean fixation count on different areas of interests (AOIs) for diabetic retinopathy photographs between ophthalmologists and optometrists.

Groups	AOI Comparison Areas		Mean Difference	*p* Value
Ophthalmologists	Disc	Macula	5.38	0.10 (NSS)
		Laser scar	10.13	<0.001 (SS)
		Hemorrhage	4.75	0.18 (NSS)
		Cotton wool spots	11.38	<0.001 (SS)
Optometrists	Disc	Macula	11.25	<0.001 (SS)
		Laser scar	11.88	<0.001 (SS)
		Hemorrhage	6.38	0.04 (SS)
		Cotton wool spots	14.13	<0.001 (SS)

SS = statistically significant; NSS = not statistically significant.

**Table 9:** Comparison of the fixation rate on different areas of interest (AOIs) between ophthalmologists and optometrists.

Areas of Interest	Fixation Rate		Mean Difference ± SD
	Ophthalmologists	Optometrists	
Optic disc (H)	2.67 ± 0.66	2.43 ± 0.51	0.24 ± 0.56 (NSS)
Optic disc (DR)	3.52 ± 0.94	3.31 ± 0.61	0.21 ± 1.25 (NSS)
Macula (H)	4.02 ± 1.05	4.18 ± 1.01	−0.16 ± 1.14 (NSS)
Macula (DR)	2.32 ± 1.99	5.06 ± 4.96	−2.74 ± 6.08 (NSS)
Retina areas	4.49 ± 1.10	4.42 ± 0.93	0.08 ± 0.50 (NSS)
Major blood vessels	4.76 ± 3.37	3.28 ± 1.71	1.48 ± 4.02 (NSS)
Lesions/abnormality area			
-laser scar	3.55 ± 2.48	4.45 ± 1.31	−0.90 ± 3.15 (NSS)
-hemorrhage	4.05 ± 1.17	3.38 ± 0.80	0.66 ± 1.66 (NSS)
-cotton wool spots	1.83 ± 2.55	1.44 ± 2.39	0.40 ± 3.99 (NSS)

H = healthy fundus images; DR = diabetic retinopathy fundus images; NSS = not statistically significant.

**Table 10:** Analysis of variance (ANOVA) for mean fixation rate on different areas of interests (AOIs) between ophthalmologists and optometrists.

Groups		df	F Value	*p* Value
Ophthalmologists	Between AOIs (H)	3	1.95	0.14 (NSS)
	Within AOIs (H)	28		
Optometrists	Between AOIs (H)	3	5.16	0.01 (SS)
	Within AOIs (H)	28		
Ophthalmologists	Between AOIs (DR)	4	1.84	0.14 (NSS)
	Within AOIs (DR)	35		
Optometrists	Between AOIs (DR)	4	2.31	0.08 (NSS)
	Within AOIs (DR)	35		

H = healthy fundus images; DR = diabetic retinopathy fundus images; SS = statistically significant.

**Table 11:** The Tukey post hoc analysis of multiple comparisons for the mean fixation rate on different areas of interests (AOIs) for healthy fundus photographs between ophthalmologists and optometrists.

Groups	AOI Comparison Areas		Mean Difference	*p* Value
Optometrists	Disc	Macula	−1.75	0.02 (SS)
		Retina Areas	−1.98	0.01 (SS)
		Major Blood Vessels	−0.85	0.45 (NSS)

SS = statistically significant; NSS = not statistically significant.

**Table 12:** Percentage of correct diagnosis score.

Fundus Photographs	Group	Min (%)	Max (%)	Mean Score ± SD (%)
Healthy	Ophthalmologists	16.67	100.00	32.29 ± 27.97
	Optometrists	<0.001	83.33	37.50 ± 27.82
Diabetic retinopathy	Ophthalmologists	66.67	100.00	81.25 ± 10.68
	Optometrists	66.67	100.00	87.50 ± 11.79
Total correct diagnosis	Ophthalmologists	50.00	83.33	66.67 ± 11.79
	Optometrists	50.00	83.33	62.50 ± 12.60

**Table 13:** Paired t-test results comparing correct diagnosis scores between ophthalmologists and optometrists.

Correct Diagnosis Score	*n*	Mean Difference ± SD	*t*	df	*p*-Value
Healthy fundus photographs	8	0.88 ± 2.03	1.22	7	0.26 (NSS)
Diabetic retinopathy fundus photographs	8	−0.38 ± 1.19	−0.89	7	0.40 (NSS)
Total correct diagnosis score	8	0.50 ± 1.77	0.80	7	0.45 (NSS)

NSS = not statistically significant.

**Table 14:** Correlation between total fixation duration, total fixation count, total fixation rate and correct diagnosis between ophthalmologists and optometrists.

	Total Fixation Duration	Total Fixation Count	Total Fixation Rate
**Correct Diagnosis (H)**	Ophthalmologists (*r* = 0.17, *p* = 0.68)	Ophthalmologists (*r* = −0.02, *p* = 0.97)	Ophthalmologists (*r* = −0.25, *p* = 0.55)
	Optometrists (*r* = −0.16, *p* = 0.71)	Optometrists (*r* = −0.48, *p* = 0.23)	Optometrists (*r* = 0.05, *p* = 0.91)
**Correct Diagnosis (DR)**	Ophthalmologists (*r* = 0.77, *p* = 0.03) ^SS^	Ophthalmologists (*r* = 0.67, *p* = 0.07)	Ophthalmologists (*r* = 0.40 *p* = 0.33)
	Optometrists (*r* = 0.35, *p* = 0.34)	Optometrists (*r* = 0.23, *p* = 0.58)	Optometrists (*r* = 0.61, *p* = 0.11)

H = healthy fundus images; DR = diabetic retinopathy fundus images; ^SS^ = statistically significant (*p* < 0.05).
